# Adverse pregnancy and perinatal outcome in patients with recurrent pregnancy loss: Multiple imputation analyses with propensity score adjustment applied to a large‐scale birth cohort of the Japan Environment and Children’s Study

**DOI:** 10.1111/aji.13072

**Published:** 2018-12-13

**Authors:** Mayumi Sugiura‐Ogasawara, Takeshi Ebara, Yasuyuki Yamada, Naoto Shoji, Taro Matsuki, Hirohisa Kano, Takahiro Kurihara, Toyonori Omori, Motohiro Tomizawa, Maiko Miyata, Michihiro Kamijima, Shinji Saitoh, the Japan Environment, Children’s Study (JECS) Group

**Affiliations:** ^1^ Department of Obstetrics and Gynecology Nagoya City University Graduate School of Medical Sciences Nagoya Japan; ^2^ Department of Occupational and Environmental Health Nagoya City University Graduate School of Medical Sciences Nagoya Japan; ^3^ Juntendo University Graduate School of Health and Sports Science Chiba Japan; ^4^ Department of Health Care Policy Management Nagoya City University Graduate School of Medical Sciences Nagoya Japan; ^5^ National Center for Child Health and Development Tokyo Japan; ^6^ Department of Pediatrics and Neonatology Nagoya City University Graduate School of Medical Sciences Nagoya Japan

**Keywords:** birth cohort study, multiple imputation analyses, perinatal outcome, pregnancy outcome, propensity score adjustment, recurrent pregnancy loss

## Abstract

**Problem:**

Several studies have reported the increased risk of preterm birth, premature rupture of membranes, and low birth weight in patients with recurrent pregnancy loss (RPL). There have been a limited number of large population‐based studies examining adverse pregnancy and perinatal outcome after RPL. Multiple‐imputed analyses (MIA) adjusting for biases due to missing data is also lacking.

**Method of study:**

A nationwide birth cohort study known as the “Japan Environment and Children’s Study (JECS)” was conducted by the Ministry of the Environment. The subjects consisted of 104 102 registered children (including fetuses or embryos).

**Results:**

No increased risk of a congenital anomaly, aneuploidy, neonatal asphyxia, or a small for date infant was observed among the children from women with a history of RPL. A novel increased risk of placental adhesion and uterine infection was found. The adjusted ORs using MIA in women with three or more PL were 1.76 (95% CI, 1.04‐2.96) for a stillbirth, 1.68 (1.12‐2.52) for a pregnancy loss, 2.53 (1.17‐5.47) for placental adhesion, 1.87 (1.37‐2.55) and 1.60 (.99‐2.57) for mild and severe hypertensive disorders of pregnancy, respectively, 1.94 (1.06‐3.55) for uterine infection, 1.28 (1.11‐1.47) for caesarean section and .86 (.76‐.98) for a male infant.

**Conclusion:**

MIA better quantified the risk, which could encourage women who might hesitate to attempt a subsequent pregnancy.

## 1 INTRODUCTION

Miscarriage is the most common pregnancy complication with a frequency of 15%.^1^, ^2^ Recurrent pregnancy loss (RPL) is defined as two or more losses at any time during pregnancy.^2^, ^3^ Most of these occur before 12 weeks of gestation.

RPL is a heterogenous reproductive problem with multiple etiologies and contributing factors include age, body mass index (BMI), parity, and smoking habit.^1^, ^2^ Identifiable causes of RPL include antiphospholipid syndrome (APS), uterine anomalies and parental and embryonic chromosomal abnormalities.^1^, ^2^, ^3^, ^4^, ^5^, ^6^ Endocrine, infectious, and immune inflammatory conditions have been reported to be associated with RPL. Of these, APS is the treatable etiology.^1^, ^2^ There has been no randomized control trial to compare the live birth rate between with and without surgery for a uterine anomaly or preimplantation genetic diagnosis for a translocation.

However, the cumulative live birth rate was 84% in non‐genetic carriers and 85.5% in couples with no explanation in the previous studies.^5^, ^7^ The live birth rate decreased significantly according to the number of previous miscarriages in both groups.^8^ Information on the adverse pregnancy and perinatal outcome is available but limited.^9^, ^10^ The risk of a preterm birth (PTB) is most frequently examined.^9^, ^10^, ^11^, ^12^, ^13^, ^14^, ^15^ Other previous studies have shown a significantly increased risk of very PTB.^13^, ^14^, ^15^, ^16^ Premature rupture of membrane (PROM),^12^, ^14^, ^17^ low birth weight (LBW),^11^, ^13^, ^15^ caesarean section, placenta abruptio, and hypertensive disorders of pregnancy (HDP).^17^

However, there has been controversy regarding whether RPL increases the risk of a congenital anomaly or neonatal asphyxia. A case‐control study with 18 534 malformed and 17 544 non‐malformed babies indicated that multiple malformations, Down’s syndrome, anencephaly, spinabifida, talipes equinovarus, congenital dislocation of the hip and LBW were associated with previous miscarriage and stillbirth.^11^ Another study with 638 recurrent miscarriages (RM) patients and 3099 non‐RM patients also reported a risk of congenital anomalies with RM.^12^ A recent study found no association between RM and congenital anomaly or aneuploidy.^10^

These studies did not employ a population‐based cohort, but rather case‐control retrospective approaches. The influence of covariates and medical histories were not considered.^11^, ^12^ Furthermore, recent concern regarding the treatment of missing data has been raised because the generalizability of findings might be limited by the extent of the missing values.

We have conducted the nationwide population‐based birth cohort study known as the “Japan Environment and Children’s Study (JECS)” planned by the Ministry of the Environment, Government of Japan.^18^, ^19^, ^20^, ^21^, ^22^ The study subjects consisted of 104 102 registered pregnancies recruited during the first 3 years of the JECS, and their babies are now being followed up for 13 years mainly to examine the influence of the uterine environment on the fetus.

We determined the adverse pregnancy and perinatal outcome according to the number of previous pregnancy losses (PL) reported by the JECS with the use of multiple imputation analyses (MIA).

## 2 METHODS

### 2.1 Study design and participants

Pregnant women were recruited by the JECS between January 31, 2011 and March 31, 2014.

Eligibility criteria for expectant mothers were as follows: that they (i) resided at the time of recruitment in any of the study areas selected by 15 Regional JECS Centers located countrywide, (ii) had an expected delivery date after August 1, 2011, and (iii) were capable of comprehending the Japanese language and completing the self‐administered questionnaire.^18^, ^19^, ^20^, ^21^, ^22^ The sample size has been calculated in the JECS protocol by the Ministry of the Environment.^23^ In principle, pregnant women completed the questionnaire during the second (MT1) and third trimester (MT2). Their medical records were transcribed by doctors or research coordinators at registration (DrT1), just after delivery (Dr0m) and at 1 month after delivery (Dr1m).

**Figure 1:**
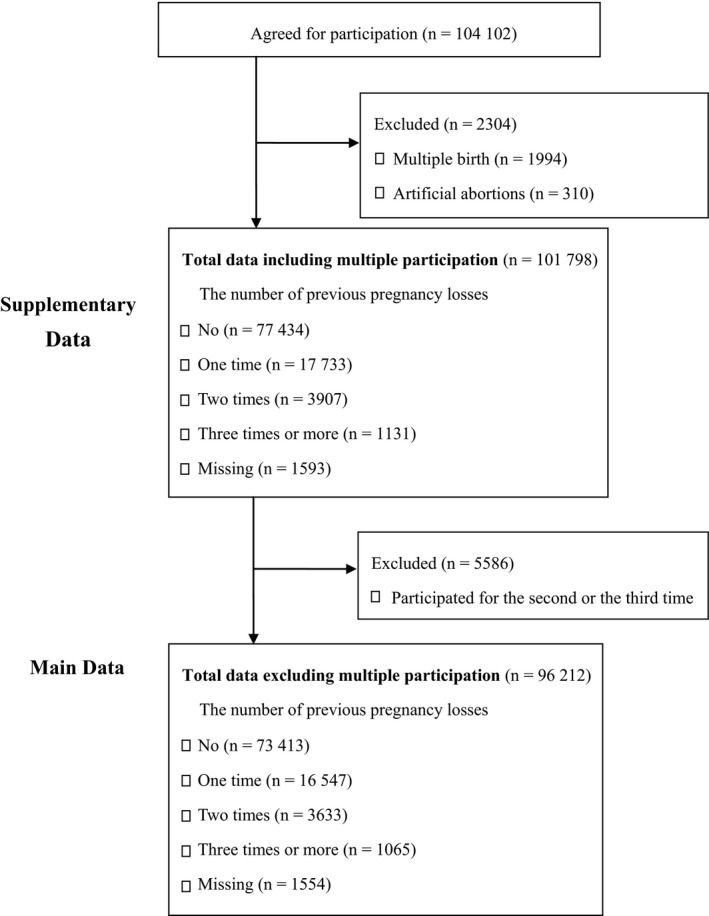
The flow diagram for assessing eligibility

The present study was based on the jecs‐ag‐20160424 dataset, which includes 104 102 registered children (including fetuses and embryos), and was released restrictively to all concerned in July, 2016 (Figure 1). A total of 1994 children of mothers with multiple pregnancies were excluded because several outcomes were influenced by multiple pregnancies. Furthermore, 310 fetuses or embryos terminated by induced abortion were also excluded. In addition, a total of 5586 children (fetuses or embryos) whose mothers had participated for the second or the third time were excluded. Finally, 96 212 participants were included in the main analysis. The mean (SD) age at registration was 30.7 (5.1). The mean (SD) gestational weeks at registration was 14.0 (5.7) weeks.

The JECS protocol was reviewed and approved by the Ministry of the Environment’s Institutional Review Board on Epidemiological Studies and by the Ethics Committees of all participating institutions. Written informed consent was obtained from all participating women.

### 2.2 Data collection

The first questionnaire (MT1) included the sociodemographic characteristics, medical histories and the details of all previous pregnancies.

Medical histories included atopic dermatitis, asthma, collagen disease, autoimmune disease, systematic lupus erythematosus (SLE), rheumatic arthritis (RA), insulin‐dependent diabetes mellitus (IDDM), non‐insulin‐dependent DM (NIDDM), gestational diabetes, hyperthyroidism, hypothyroidism, anemia, hypertension, hyperlipidemia, stroke, myocardiac infarction, congenital heart disease, Kawasaki disease, depression, dysautonomia, anxiety disorder, gastroesophageal reflex disease, gastritis, gastric ulcer, duodenal ulcer, irritable colon, Crohn’s disease, ulcerative colitis, fatty liver, chronic nephritis, nephrotic syndrome, menstrual disorder, endometriosis, adenomyosis, uterine fibroids, uterine anomaly, ovarian tumor, and polycystic ovary syndrome (PCOS).

The socioeconomic status was assessed by the education level and annual household income in the second questionnaire (MT2).

The first medical record transcript (DrT1) included maternal age, gestational weeks at registration, maternal body weight, height, conception, and the details of all previous pregnancies (vaginal delivery/caesarian delivery/miscarriage/induced abortion/stillbirth).

The Dr0m included maternal age, gestational weeks at miscarriage and delivery, single/multiple, live birth/stillbirth, miscarriage/induced abortion, male/female, birth weight, vaginal/caesarian delivery, pregnancy complications, and perinatal outcomes.

The third medical record transcription (Dr1m) included questions on the presence/absence of congenital anomalies.

### 2.3 Outcome, exposure, and covariates

The pregnancy histories provided by the doctors in filling out the DrT1 form were given priority over the participants’ answers with regard to the number of previous PL (categorized as 0, 1, 2, 3 or more).

The obstetric outcomes included stillbirth > 20 weeks’ gestation, late miscarriage, early miscarriage <12 weeks’ gestation, PL, PTB (<37 and <34 weeks’ gestation), PROM, placenta praevia, abruptio placenta, adherent placenta, oligohydramnios, mild and severe HDP, uterine infection and caesarean section. The perinatal outcomes were small‐for‐date of the 10th percentile (SFD), IUFD, sex, Apgar score <7 at 5 minutes, an umbilical artery blood pH <7.1, the presence/absence of congenital anomalies of the head, eyes, ears, face, limbs, lungs, heart, intestine, urogenital organs, skin, and skeleton and chromosome aneuploidy. The presence of chromosomal aneuploidy as indicated by the doctors in filling out the Dr1m was given priority over the Dr0m.

Potential covariates were maternal age at registration, BMI, marital status, the presence/absence of IVF‐ET, previous live birth, smoking, education, and income.

### 2.4 Statistical analysis

The associations between the number of previous PL and covariates were tested using chi‐squared tests for categorical variables. Associations between each outcome and the covariates were examined using Fisher’s exact test.

The prevalence of each outcome including the crude odds ratio (OR) and the 95% confidence interval (CI) were calculated for 0 (reference), 1, 2, and 3 or more PL (as categorized). We conducted both complete‐case analysis and MIA using logistic regression to estimate the risks of previous PL affecting the adverse pregnancy and perinatal outcome. In the complete‐case analysis, the unadjusted model (Model I) was used to estimate the independent effects of the PL history, and the adjusted model (Model II) included potential confounders in the model, adjusting for the propensity score including all covariates with *P* values <.05.^24^, ^25^ In general, it has been found that propensity score adjustment has the advantages of being less biased, providing more robust estimates than those of the traditional logistic regression. An unlimited number of covariates can be theoretically included in propensity score estimation so that propensity score adjustment has been employed when adjusting for a large number of covariates and widely used in recent observational research.

Adjusted Model III in accord with Model II included MIA. Since the percentage of missing values in Model II across all variables ranged from 5.2% to 21.1%, prior to conducting logistic regression analyses using propensity score adjustment, MIA was applied to handle missing data. Five imputed datasets were created for each outcome, and all variables that were used in Model IIs were included in each imputation model using the propensity score. Use of MIA is more efficient in most settings and widely recommended for improving biases within complete‐case analyses.^26^ Model IV additionally adjusted for covariates related to the gynecological history in Model III. It was speculated that women with PL could have a greater chance of being diagnosed with a gynecological disease as a result of frequent ultrasound sonography. Thus, we performed MIA with and without covariates for gynecological history.

All calculations were carried out using SPSS version 23 and 24 (IBM Corp., Japan).

## 3 RESULTS

Histories of previous PL were available for 94 658 participants out of 96 212. Among these women, 77.6% (73 413) had no history of PL, 17.5% (16 547) had experienced one PL, 3.8% (3633) experienced two losses, and 1.1% (1065) experienced three or more losses (Table 1). Age, BMI, IVF‐ET and previous live births were positively associated with the number of previous PL. The rates of marital status, smoking, education background, and income were influenced by the number of previous PL. All the listed variables were significantly associated with an increasing number of previous PL (Table 1, *P *< .0001 for all covariates). In the following analyses, women with no PL were regarded as the reference category.

**Table 1:** Demographic characteristics of women according to the number of previous pregnancy losses (n = 94 658)

Variables	No miscarriage (n = 73 413)		One (n = 16 547)		Two (n = 3633)		Three or more (n = 1065)		*P*‐value
Age (years), % (n)									<0.0001
<20	1.5	(1026)	0.3	(52)	0.2	(7)	0.3	(3)	
20‐29	42.8	(30 278)	29.9	(4,758)	21.6	(759)	15.5	(158)	
30‐39	52.9	(37 392)	64.4	(10,264)	68.1	(2,388)	67.9	(694)	
>40	2.8	(1971)	5.4	(859)	10.1	(354)	16.3	(167)	
Missing, % (n)	3.7	(2746)	3.7	(614)	3.4	(125)	4.0	(43)	
BMI, % (n)									<0.0001
<18.5	16.7	(12 264)	14.8	(2,442)	14.3	(519)	14.5	(154)	
18.5‐25.0	72.9	(53 368)	74.0	(12,212)	73.1	(2,651)	72.7	(773)	
≥25.0	10.4	(7587)	11.2	(1,852)	12.6	(455)	12.9	(137)	
Missing, % (n)	0.3	(194)	0.2	(41)	0.2	(8)	0.1	(1)	
Marital status, % (n)									<0.0001
Married	95.0	(67 516)	97.1	(15,677)	97.5	(3,457)	96.4	(1,004)	
Single	4.3	(3068)	1.7	(282)	1.0	(34)	0.9	(9)	
Divorced	0.7	(518)	1.2	(190)	1.6	(55)	2.7	(28)	
Missing, % (n)	3.1	(2311)	2.4	(398)	2.4	(87)	2.3	(24)	
IVF‐ET, % (n)									
Carried out	2.7	(1965)	4.2	(702)	5.2	(188)	9.4	(100)	
Missing, % (n)	0.0	(20)	0.0	(4)	0.0	(1)	0.0	(0)	
Previous live birth, % (n)									
Yes	52.1	(38 258)	68.8	(11,354)	76.7	(2,781)	79.0	(840)	<0.0001
Missing, % (n)	0.0	(18)	0.3	(49)	0.2	(8)	0.2	(2)	
Smoking, % (n)									<0.0001
Never smoked	59.4	(42 128)	55.6	(8,952)	53.0	(1,878)	52.4	(543)	
Quit smoking before pregnancy	22.3	(15 798)	26.8	(4,311)	26.3	(931)	29.7	(308)	
Quit smoking during early pregnancy	13.8	(9774)	12.2	(1,969)	13.5	(478)	11.1	(115)	
Current smoker	4.5	(3198)	5.3	(860)	7.2	(254)	6.8	(71)	
Missing, % (n)	3.4	(2515)	2.7	(455)	2.5	(92)	2.6	(28)	
Educational background (years), % (n)									<0.0001
Junior high/ High school	35.7	(24 974)	36.7	(5,809)	39.2	(1,356)	42.1	(426)	
College/Junior college/Technology college	41.8	(29 258)	43.1	(6,829)	42.3	(1,465)	41.8	(423)	
University	20.9	(14 613)	18.8	(2,981)	17.5	(607)	15.0	(152)	
Graduate school	1.5	(1079)	1.4	(214)	1.0	(35)	1.0	(10)	
Missing, % (n)	4.8	(3489)	4.3	(714)	4.7	(170)	5.1	(54)	
Income (JPY), % (n)									<0.0001
<200	5.7	(3720)	5.4	(802)	5.2	(168)	6.7	(64)	
200‐< 400	34.9	(22 736)	33.2	(4,943)	31.9	(1,031)	30.5	(292)	
400‐< 600	32.8	(21 382)	33.7	(5,016)	32.9	(1,065)	33.9	(324)	
600‐< 800	15.8	(10 269)	16.4	(2,446)	18.1	(586)	16.4	(157)	
800‐< 1,000	6.6	(4295)	6.6	(984)	7.0	(228)	8.3	(79)	
≥1,000	4.2	(2728)	4.6	(684)	4.9	(158)	4.3	(41)	
Missing, % (n)	11.3	(8283)	10.1	(1,672)	10.9	(397)	10.1	(108)	

BMI: Body Mass Index, IVF‐ET: In Vitro Fertilization‐Embryo Transfer, JPY: ×10 000 Japanese yen, 1US$ = 103JPY, as of September, 2016, *P*‐value: Fisher’s exact test.

* Histories of pregnancy losses were not available for 1554 participants out of 96 212.

### 3.1 Model I (crude analysis)

Crude analysis showed a significant association with stillbirth, PL, PTB (both <37 and <34 weeks’ gestation), placental adhesion, HDP, caesarean section and a male infant with three or more PL (Model I in Tables 2 and 3). There was no risk associated with PROM, placenta praevia, oligohydramnios, abruptio placenta, or uterine infection. No association with SFD, IUFD, LBW, low Apgar score, low pH, congenital anomaly, or aneuploidy was observed.

**Table 2:** Obstetric outcome of women according to the number of previous pregnancy losses

	Number of pregnancy losses	Prevalence %, (n)		OR, 95%CI				Pooled OR, 95%CI				Covariates included in propensity score
				Model I		Model II		Multiple imputation model III		Multiple imputation model Ⅳ		
Late miscarriage and stillbirth> 20 weeks gestation	No	0.7	(534/71708)	1		1		1		1		Age, BMI, Smoking, ART, Previous live birth, NIDDM, Gastroesophageal reflux disease, Gastric ulcer, Adenomyosis
	One	1.0	(158/16 233)	**1.31**	**1.10‐1.57**	**1.27**	**1.03‐1.57**	1.18	0.99‐1.42	1.18	0.99‐1.41	
	Two	1.2	(42/3564)	**1.59**	**1.16‐2.18**	**1.49**	**1.03‐2.15**	1.34	0.97‐1.84	1.33	0.97‐1.82	
	Three	1.6	(17/1034)	**2.23**	**1.37‐3.63**	**2.12**	**1.23‐3.65**	**1.76**	**1.04‐2.96**	**1.72**	**1.05‐2.83**	
	Missing, % (n)			3.8	(3673)	9.7	(9332)	2.8	(2646)	2.8	(2646)	
Early miscarriage<12 weeks gestation	No	0.3	(238/71 412)	1		1		1		1		Age, Ulcerative colitis
	One	0.5	(76/16 151)	**1.41**	**1.09‐1.83**	**2.09**	**1.34**‐**3.23**	**1.38**	**1.05‐1.81**	**1.38**	**1.05‐1.81**	
	Two	0.3	(9/3531)	0.76	0.39‐1.49	1.11	0.40‐3.08	0.68	0.35‐1.33	0.68	0.35‐1.33	
	Three	0.7	(7/1024)	2.06	0.97‐4.38	2.63	0.81‐8.50	1.89	0.89‐4.02	1.89	0.89‐4.02	
	Missing, % (n)			4.3	(4094)	9.3	(8927)	3.2	(3052)	3.2	(3052)	
Pregnancy loss	No	1.1	(772/71946)	1		1		1		1		Age, BMI, Smoking, ART, Previous live birth, NIDDM, Adenomyosis
	One	1.4	(234/16 309)	**1.34**	**1.16‐1.56**	**1.38**	**1.14**‐**1.67**	**1.21**	**1.04**‐**1.41**	**1.21**	**1.05‐1.40**	
	Two	1.4	(51/3573)	**1.34**	**1.00‐1.78**	**1.43**	**1.01‐2.02**	1.09	0.82**‐**1.46	1.08	0.81‐1.44	
	Three	2.3	(24/1041)	**2.18**	**1.44‐3.28**	**2.20**	**1.34‐3.60**	**1.68**	**1.12‐2.52**	**1.73**	**1.14‐2.62**	
	Missing, % (n)			3.5	(3343)	9.6	(9238)	2.4	(2282)	2.4	(2282)	
Preterm birth<37 weeks gestation	No	4.8	(3419/71 356)	1		1		1		1		Age, Marital status, BMI, Smoking, Education, (Household income), ART, Atopic Dermatitis, Collagen disease, SLE, RA, IDDM, NIDDM, Gestational diabetes, High blood pressure, Congenital heart disease, Depression, Dysautonomia, Anxiety disorders, Gastric ulcer, Chronic nephritis, Endometriosis, Uterine fibroids, Adenomyosis, Congenital uterine anomalies
	One	5.0	(810/16 126)	1.05	0.97**‐**1.14	0.96	0.88**‐**1.05	0.98	0.90**‐**1.06	0.97	0.90**‐**1.05	
	Two	5.8	(206/3532)	**1.23**	**1.07**‐**1.42**	1.01	0.85‐1.19	1.09	0.94‐1.27	1.07	0.93‐1.24	
	Three	7.1	(73/1025)	**1.52**	**1.20**‐**1.94**	1.23	0.94‐1.61	**1.29**	**1.01‐1.65**	1.27	0.99‐1.62	
	Missing, % (n)			4.3	(4173)	17.5	(16 866)	3.3	(3164)	3.3	(3164)	
Preterm birth <34 weeks gestation	No	1.1	(793/71 356)	1		1		1		1		Age, Marital status, BMI, Smoking, Education, ART, SLE, IDDM, NIDDM, Gestational diabetes, High blood pressure, Gastric ulcer, Fatty liver, Chronic nephritis, Endometriosis, Uterine fibroids, Adenomyosis, PCOS
	One	1.3	(207/16 126)	1.16	1.00‐1.36	1.06	0.89‐1.27	1.05	0.90‐1.23	1.05	0.90**‐**1.23	
	Two	1.6	(57/3532)	**1.46**	**1.11‐1.91**	1.12	0.81**‐**1.56	1.22	0.93**‐**1.61	1.21	0.92**‐**1.60	
	Three	2.0	(21/1025)	**1.86**	**1.20‐2.88**	1.17	0.67**‐**2.05	1.43	0.92**‐**2.23	1.41	0.90**‐**2.21	
	Missing, % (n)			4.3	(4173)	11.8	(11 313)	3.3	(3164)	3.3	(3 164)	
Premature rupture of membrane	No	9.3	(6622/71 392)	1		1		1		1		Age, Marital status, Smoking, Education, (Household income), ART, Previous live birth, SLE, IDDM, Anemia, Congenital heart disease, Uterine fibroids, Adenomyosis
	One	8.2	(1315/16 131)	**0.87**	**0.82‐0.92**	0.95	0.89**‐**1.02	0.96	0.90**‐**1.02	0.96	0.90**‐**1.02	
	Two	8.2	(291/3533)	**0.88**	**0.78**‐**0.99**	1.06	0.93‐1.21	1.03	0.91‐1.17	1.03	0.91‐1.17	
	Three	8.4	(86/1025)	0.90	0.71‐1.10	1.07	0.84‐1.36	1.11	0.89‐1.39	1.12	0.89‐1.41	
	Missing, % (n)			4.3	(4131)	17.5	(16 828)	3.2	(3120)	3.2	(3120)	
Placenta praevia	No	0.6	(438/71 392)	1		1		1		1		Age, Household income, ART, Gestational diabetes, Endometriosis, Uterine fibroids, Ovarian tumors
	One	0.8	(137/16 131)	**1.39**	**1.14‐1.68**	**1.25**	**1.02**‐**1.53**	**1.26**	**1.04**‐**1.53**	**1.24**	**1.02‐1.51**	
	Two	1.1	(40/3533)	**1.86**	**1.34‐2.57**	**1.44**	**1.01‐2.06**	**1.56**	**1.12‐2.17**	**1.50**	**1.08‐2.09**	
	Three	1.1	(11/1025)	1.76	0.96‐3.21	1.30	0.69**‐**2.46	1.34	0.73**‐**2.46	1.27	0.70**‐**2.34	
	Missing, % (n)			4.3	(4131)	16.4	(15 814)	3.2	(3120)	3.2	(3120)	
Oligohydramnios	No	1.3	(944/71 392)	1		1		1		1		Marital status, BMI, (Household income), ART, Previous live birth, Gestational diabetes, Depression, Bronchial asthma
	One	1.3	(207/16 131)	0.97	0.83**‐**1.13	1.06	0.90‐1.25	1.08	0.93**‐**1.26	1.08	0.93**‐**1.26	
	Two	1.4	(50/3533)	1.07	0.80‐1.43	1.29	0.95‐1.75	1.27	0.95‐1.69	1.27	0.95‐1.69	
	Three	1.6	(16/1025)	1.18	0.72‐1.95	1.22	0.69‐2.18	1.48	0.90‐2.44	1.48	0.90‐2.44	
	Missing, % (n)			4.3	(4131)	13.8	(13 249)	3.2	(3120)	3.2	(3120)	
Abruptio placentae	No	0.4	(292/71 392)	1		1		1		1		Age, Smoking, ART, Adenomyosis
	One	0.5	(84/16 131)	**1.28**	**1.00‐1.63**	1.15	0.89‐1.49	1.21	0.95‐1.55	1.19	0.93**‐**1.53	
	Two	0.5	(18/3533)	1.25	0.77‐2.01	1.05	0.63**‐**1.75	1.13	0.70**‐**1.83	1.13	0.70**‐**1.83	
	Three	0.7	(7/1025)	1.67	0.79‐3.55	1.30	0.57**‐**2.93	1.47	0.69‐3.14	1.41	0.66‐3.00	
	Missing, % (n)			4.3	(4131)	9.8	(9419)	3.2	(3120)	3.2	(3120)	
Placental adhesion	No	0.2	(150/71 392)	1		1		1		1		Age, ART, Hyperthyroidism, Endometriosis, Adenomyosis, Congenital uterine anomalies, PCOS
	One	0.3	(45/16 131)	1.33	0.95**‐**1.86	1.20	0.84**‐**1.70	1.23	0.88**‐**1.72	1.17	0.84**‐**1.64	
	Two	0.4	(15/3533)	**2.03**	**1.19**‐**3.45**	**1.83**	**1.07‐3.14**	**1.74**	**1.01‐2.98**	1.64	0.95‐2.82	
	Three	0.7	(7/1025)	**3.27**	**1.53**‐**6.99**	**2.69**	**1.24**‐**5.82**	**2.53**	**1.17**‐**5.47**	**2.33**	**1.08**‐**5.04**	
	Missing, % (n)			4.3	(4131)	9.1	(8767)	3.2	(3120)	3.2	(3120)	
Hypertensive disorders of pregnancy (mild)	No	2.3	(1659/71 392)	1		1		1		1		Age, BMI, Smoking, Education, (Household income), ART, Previous live birth, Bronchial asthma, IDDM, NIDDM, Gestational diabetes, Hyperthyroidism, Anemia, High blood pressure, Fatty liver, Chronic nephritis, Uterine fibroids, Adenomyosis
	One	2.2	(354/16 131)	0.94	0.84**‐**1.06	0.95	0.84‐1.07	0.94	0.84‐1.06	0.95	0.84**‐**1.06	
	Two	2.5	(90/3533)	1.10	0.89**‐**1.36	1.09	0.86**‐**1.37	1.10	0.88**‐**1.36	1.10	0.88**‐**1.36	
	Three	4.3	(44/1025)	**1.89**	**1.39‐2.56**	**2.02**	**1.46‐2.78**	**1.87**	**1.37‐2.55**	**1.88**	**1.38‐2.56**	
	Missing, % (n)			4.3	(4131)	17.3	(16 626)	3.2	(3120)	3.2	(3120)	
Hypertensive disorders of pregnancy (severe)	No	1.0	(695/71 392)	1		1		1		1		Age, BMI, ART, Previous live birth, NIDDM, Anemia, High blood pressure, High cholesterol, Fatty liver, Chronic nephritis, Uterine fibroids, Congenital uterine anomalies, PCOS
	One	1.1	(171/16 131)	1.09	0.92**‐**1.29	0.96	0.80**‐**1.14	1.04	0.88**‐**1.23	1.03	0.87**‐**1.22	
	Two	0.9	(32/3533)	0.93	0.65‐1.33	0.75	0.53‐1.08	0.84	0.59‐1.21	0.85	0.60‐1.22	
	Three	1.8	(18/1025)	**1.82**	**1.13**‐**2.92**	1.29	0.80‐2.08	1.60	0.99‐2.57	**1.63**	**1.00**‐**2.64**	
	Missing, % (n)			4.3	(4131)	9.2	(8892)	3.2	(3120)	3.2	(3120)	
Uterine infection	No	0.7	(535/71 980)	1		1		1		1		Marital status, BMI, Education, ART, Previous live birth, Endometriosis
	One	0.7	(114/16 312)	0.94	0.77**‐**1.15	1.06	0.85‐1.33	1.11	0.90‐1.36	1.11	0.90**‐**1.36	
	Two	0.8	(29/3574)	1.09	0.75**‐**1.59	1.26	0.83**‐**1.93	1.42	0.97**‐**2.07	1.40	0.96**‐**2.04	
	Three	1.1	(11/1041)	1.43	0.78**‐**2.60	1.62	0.80**‐**3.28	**1.94**	**1.06‐3.55**	**1.96**	**1.07‐3.59**	
	Missing, % (n)			3.4	(3305)	7.7	(7405)	2.3	(2242)	2.3	(2242)	
Caesarean section	No	18.2	(12 978/71 126)	1		1		1		1		Age, Marital status, BMI, Smoking, Education, (Household income), ART, Atopic Dermatitis, *Bronchial asthma*, *Collagen disease*, SLE, RA, IDDM, NIDDM, Gestational diabetes, Hypothyroidism, High blood pressure, Stroke, Congenital heart disease, *Depression*, Anxiety disorders, *Gastroesophageal reflux disease*,* Gastritis*, Gastric ulcer, *Duodenal ulcer*, Fatty liver, Chronic nephritis, Endometriosis, Uterine fibroids, Adenomyosis, Congenital uterine anomalies,*Ovarian tumors*
	One	20.4	(3278/16 081)	**1.15**	**1.10‐1.20**	1.04	0.99‐1.09	1.03	0.99**‐**1.08	1.03	0.98**‐**1.07	
	Two	23.8	(840/3524)	**1.40**	**1.30**‐**1.52**	**1.13**	**1.04**‐**1.24**	**1.17**	**1.08‐1.27**	**1.15**	**1.06‐1.25**	
	Three	27.2	(277/1019)	**1.67**	**1.46**‐**1.92**	**1.31**	**1.13**‐**1.52**	**1.28**	**1.11‐1.47**	**1.25**	**1.08‐1.44**	
	Missing, % (n)			4.6	(4462)	17.8	(17 089)	3.6	(3460)	3.6	(3460)	

Model I Unadjusted model.

Model II Adjusted model using propensity scores based on complete case analysis.

Model III Adjusted model using propensity scores based on multiple imputation model.

Model IV Covariates about gynecological history (underlined covariates) were added to the model III.

Values in boldface indicated a statistical significance **level of 0.05.**

OR: Odds Ratio, 95%CI: 95% Confidence Interval.

Household income ( ) was not included in the multiple‐imputation model.

Disorders in *italic *were excluded from Model III and Model IV.

**Table 3:** Perinatal outcome of children according to the number of previous pregnancy losses

	Number of pregnancy losses	Prevalence %, (n)		OR, 95%CI					Pooled OR, 95%CI				Covariates included in propensity score
				Model I			Model II		Multiple imputation model III		Multiple imputation model Ⅳ		
SFD	No	10.3	(7,370/71,271)		1		1		1		1		Age, Marital status, BMI, Smoking, Previous live birth, Collagen disease, SLE, IDDM, Anemia, High blood pressure, Congenital heart disease, Adenomyosis, PCOS
	One	9.2	(1,486/16,106)		**0.88**	**0.83‐0.93**	0.97	0.91‐1.03	0.96	0.91‐1.02	0.96	0.91**‐**1.02	
	Two	8.7	(306/3,528)		**0.82**	**0.73‐0.93**	0.95	0.84**‐**1.07	0.96	0.85**‐**1.08	0.96	0.85**‐**1.09	
	Three	8.6	(88/1,025)		0.81	0.65**‐**1.01	0.99	0.78**‐**1.24	0.98	0.79**‐**1.23	1.01	0.81**‐**1.26	
	Missing, % (n)				4.5	(4,282)	10.5	(10,124)	3.4	(3,273)	3.4	(3,273)	
IUFD	No	0.4	(294/71,980)		1		1		1		1		Age, BMI, Adenomyosis
	One	0.5	(80/16,312)		1.20	0.94**‐**1.54	1.11	0.86**‐**1.43	1.10	0.86**‐**1.42	1.11	0.86**‐**1.42	
	Two	0.4	(16/3,574)		1.10	0.66‐1.82	0.95	0.57‐1.58	0.94	0.56‐1.56	0.92	0.55‐1.53	
	Three	0.8	(8/1,041)		1.89	0.93‐3.82	1.53	0.75‐3.12	1.55	0.76‐3.16	1.53	0.76‐3.11	
	Missing, % (n)				3.4	(3,305)	7.2	(6,965)	2.3	(2,242)	2.3	(2,242)	
Low birth weight<2500g	No	8.5	(6,051/71,300)		1		1		1		1		Age, Marital status, BMI, Smoking, Education, ART, Previous live birth, Collagen disease, SLE, RA, NIDDM, High blood pressure, Congenital heart disease, Depression, Anxiety disorders, Chronic nephritis, Nephrotic syndrome, Endometriosis, Uterine fibroids, Adenomyosis, Congenital uterine anomalies
	One	7.9	(1,274/16,115)		**0.93**	**0.87‐0.99**	**0.92**	**0.86‐0.98**	**0.93**	**0.87‐0.99**	**0.92**	**0.86‐0.98**	
	Two	9.1	(321/3,529）		1.08	0.96**‐**1.21	1.04	0.91**‐**1.18	1.09	0.97**‐**1.23	1.06	0.95**‐**1.20	
	Three	10.0	(102/1,024)		1.19	0.97‐1.47	1.18	0.95**‐**1.47	1.20	0.98‐1.47	1.17	0.95‐1.45	
	Missing, % (n)				4.4	(4,244)	11.9	(11,438)	3.4	(3,236)	3.4	(3,236)	
Male	No	51.3	(36,804/71,700)		1		1		1		1		Gestational diabetes, Dysautonomia
	One	51.8	(8,402/16,219)		1.02	0.98‐1.05	1.02	0.98**‐**1.05	1.02	0.99**‐**1.06	1.02	0.99**‐**1.06	
	Two	51.8	(1,844/3,557)		1.02	0.95‐1.09	1.02	0.95‐1.09	1.02	0.95‐1.09	1.02	0.95‐1.09	
	Three	47.7	(493/1,034)		**0.86**	**0.76‐0.98**	**0.87**	**0.77‐0.98**	**0.86**	**0.76‐0.98**	**0.86**	**0.76‐0.98**	
	Missing, % (n)				3.8	(3,702)	5.2	(4,987)	2.8	(2,676)	2.8	(2,676)	
Apgar score <7 at 5 minute	No	0.6	(419/67,588)		1		1		1		1		Age, BMI, Smoking, ART, Previous live birth, Atopic Dermatitis, IDDM, NIDDM, High blood pressure, Crohn's disease, Uterine fibroids, Adenomyosis
	One	0.8	(117/15,337)		**1.23**	**1.00‐1.51**	**1.24**	**1.00‐1.54**	1.20	0.98‐1.49	1.19	0.97‐1.47	
	Two	0.7	(22/3,364)		1.06	0.69**‐**1.62	1.05	0.67**‐**1.64	1.00	0.64**‐**1.54	1.00	0.65**‐**1.55	
	Three	0.5	(5/978)		0.82	0.34**‐**1.99	0.51	0.16**‐**1.59	0.76	0.31**‐**1.84	0.74	0.31**‐**1.81	
	Missing, % (n)				9.3	(8,945)	14.7	(14,184)	8.3	(8,015)	8.3	(8,015)	
pH<7.1	No	1.2	(697/59,523)		1		1		1		1		ART, Previous live birth, NIDDM, Anemia, Crohn's disease
	One	1.2	(166/13,545)		1.05	0.88**‐**1.24	1.16	0.98‐1.38	1.16	0.98‐1.39	1.16	0.98‐1.39	
	Two	1.2	(36/2,947)		1.04	0.75‐1.46	1.22	0.86‐1.72	1.22	0.87‐1.71	1.22	0.87‐1.71	
	Three	1.1	(10/871)		0.98	0.52‐1.84	1.10	0.57‐2.13	1.20	0.64‐2.27	1.20	0.64‐2.27	
	Missing, % (n)				20.1	(19,326)	21.1	(20,337)	19.3	(18,587)	19.3	(18,587)	
Congenital anomaly	No	12.2	(8,607/70,321)		1		1		1		1		Age, BMI, Education, (Household income), ART, Autoimmune disease, IDDM, Gestational diabetes, Hyperthyroidism, Hypothyroidism, High blood pressure, Congenital heart disease, Depression, Gastritis, Irritable bowel syndrome, Uterine fibroids, Adenomyosis
	One	12.6	(2,004/15,899)		1.03	0.98**‐**1.09	1.01	0.95‐1.06	1.01	0.96‐1.06	1.01	0.96**‐**1.06	
	Two	13.2	(461/3,492)		1.09	0.99**‐**1.21	1.01	0.91**‐**1.13	1.04	0.94**‐**1.15	1.04	0.94**‐**1.15	
	Three	12.7	(129/1,015)		1.04	0.87**‐**1.26	0.97	0.80**‐**1.18	0.96	0.80**‐**1.16	0.96	0.80**‐**1.16	
	Missing, % (n)				5.7	(5,485)	17.8	(17,142)	4.7	(4,505)	4.7	(4,505)	
Aneuploidy	No	0.2	(140/71,424)		1		1		1		1		Age, Smoking, Previous live birth, Gestational diabetes, Depression, Uterine fibroids, Adenomyosis
	One	0.2	(38/16,136)		1.20	0.84**‐**1.72	0.94	0.65**‐**1.37	0.99	0.69**‐**1.42	0.97	0.67**‐**1.39	
	Two	0.2	(8/3,533)		1.16	0.57‐2.36	0.73	0.34‐1.58	0.80	0.39‐1.64	0.82	0.40‐1.68	
	Three	0.2	(2/1,025)		1.00	0.25‐4.03	0.63	0.16‐2.58	0.61	0.15‐2.46	0.59	0.15‐2.31	
	Missing, % (n)				4.3	(4,094)	9.8	(9,451)	3.2	(3,083)	3.2	(3,083)	

Model I Unadjusted model.

Model II Adjusted model using propensity scores based on complete case analysis.

Model III Adjusted model using propensity scores based on multiple imputation model.

Model IV Covariates about gynecological history (underlined covariates) were added to the model III.

Values in boldface indicated a statistical significance level of 0.05.

OR, Odds Ratio; 95%CI, 95% Confidence Interval; SFD, Small for date; IUFD, Intrauterine fetal death.

Household income ( ) was not included in the multiple‐imputation model**.**

### 3.2 Model II (the adjustment of multiple covariates)

After the adjustment of multiple covariates with significance for each outcome, stillbirth, PL, placental adhesion, HDP, caesarean section and a male infant remained significantly associated with three or more pregnancy losses (Model II in Tables 2 and 3). The significance of PTB (both at <37 and <34 weeks’ gestation) disappeared after the adjustment.

### 3.3 Model III (the adjustment with MIA)

After the adjustment with MIA, a novel increased risk of placental adhesion and uterine infection was found (Model III in Table 2). ORs of placental adhesion increased significantly according to the number of previous PL. ORs of uterine infection tended to increase with the number of pregnancy losses and statistical significance was found in the ORs of three pregnancy losses. No increased risk of adverse perinatal outcomes such as a congenital anomaly, aneuploidy, neonatal asphyxia or SFD was observed (Model III in Tables 3). Adjusted OR with MIA were as follows: 1.76 (95% CI, 1.04‐2.96) for stillbirth, 1.68 (1.12‐2.52) for PL, 1.29 (1.01‐1.65) for PTB <37 weeks, 2.53 (1.17‐5.47) for placental adhesion, 1.87 (1.37‐2.57) and 1.60 (.99‐2.57) for mild and severe HDP, 1.94 (1.06‐3.55) for uterine infection, 1.28 (1.11‐1.47) for caesarean section, and .86 (.76‐.98) for a male infant in women with a history of three or more PL (Model III, Tables 2 and 3). Regarding stillbirth, ORs tended to be increased and was significantly higher with three pregnancy losses. With regard to HDP and caesarean section, the prevalence for women with three pregnancy losses was significantly higher. Marginally increased risks were 1.18 (.96‐1.46) for LBW. There was no risk of a very PTB <34 weeks, PROM, placenta praevia, oligohydramnios or abruptio placentae after adjustment. As for placenta praevia, the prevalence tended to increase, but that of women with three pregnancy losses was not significant.

### 3.4 Model IV (the adjustment with MIA including gynecological histories)

Similar results were obtained with MIA including covariates for gynecological history (Model IV, Tables 2 and 3).

The risk of placental adhesion and uterine infection was analyzed according to the number of induced abortions. The results were similar to those in RPL (Table S1).

### 3.5 Analysis including multiple participants

In another supplementary analysis to assess the risk as it appears in a clinical setting where patients present with a range of pregnancy histories, we incorporated 5586 children whose mothers participated in the JECS for the second or third time. When a total of 101 798 participants were analyzed, 17.7% (17 733) had experienced one PL, 3.9% (3907) experienced two losses, 1.1% (1131) experienced three or more losses and 77.3% (77 434) had no history of PL (100 205 participants whose data about pregnancy loss were available, missing, 1593, Table S2). ORs for rare outcomes displaying marginally significant increases in Tables 2 and 3 yielded statistical significance. Adjusted OR with MIA were as follows: 1.36 (1.08 to 1.71) for PTB <37 weeks’ gestation, 1.61 (1.00‐2.58) for oligohydramnios, 1.99 (1.05‐3.79) for abruptio placenta and 1.23 (1.01‐1.51) for a LBW (Model III, Tables S3 and S4).

## 4 DISCUSSION

The present study revealed no risk of a congenital anomaly, aneuploidy, neonatal asphyxia, or SFD related to RPL.

The absence of RPL effect on congenital anomalies concurs with the results in RM reported in the PROMISE trial.^27^ Our results showing the lack of risk of the aneuploidy were not attributable to noninvasive prenatal testing (NIPT) or preimplantation genetic test of aneuploidy (PGT‐A). NIPT is permitted only for research purposes by the Japanese Association of Medical Sciences. RPL does not meet NIPT criteria. In addition, PGT‐A is prohibited by the Japan Society of Obstetrics and Gynecology for ethical reasons.

We found a novel significant association between RPL and uterine infection and placental adhesion. The risk might be due not to RPL pathology but to surgery because the results of analysis according to the number of induced abortions were similar to those in RPL (Table S1) and 79.4% of facilities use curettage at induced abortion in Japan.^28^ Surgical management of the miscarriage using curettage is mainly selected.

The supplementary analysis including participants for the second or third time revealed increased risk of PTB <37 weeks’ gestation, oligohydramnios, abruptio placenta, and LBW. A recent retrospective study comparing 2030 patients with RM and 28 023 participants with no RM showed an increased risk of PTB and perinatal death after adjustment of covariates but no significantly increased risk of LBW, low Apgar score, congenital anomalies, or aneuploidy.^10^ A historical study with 732 719 nulliparous women who had a first live birth showed that women with RM were at the greatest risk (adjusted OR 1.73; 95%CI 1.57‐1.90) and the greatest association was with extreme PTB (24‐28 weeks, adjusted OR 3.87; 95%CI, 2.85‐5.26).^16^

The present study found no association with a PTB <34 weeks or PROM after MIA in contrast with previous studies.^12^, ^13^, ^14^, ^15^, ^16^, ^17^ Further study with the use of MIA is necessary to confirm this association.

HDP and caesarean section were both associated with RPL. This is in line with the results of a population‐based study with 154 294 women, which indicated an increased risk of caesarean section, placenta abruptio, and hypertensive disorder after two or more miscarriages.^17^ Recently, 472 variants in 187 genes have been reported to be associated with RPL. A meta‐analysis revealed a significant association between RM and 21 genetic variants with ORs .51‐2.37.^29^ Common risk alleles such as annexin A5 might influence both HDP and RPL.^30^, ^31^

Finally, women with three or more PL had a lower tendency to have a male infant on the index pregnancy

A previous study proved that boys were significantly more common than girls among births prior to a secondary RM and the chance of a live birth after RM is lower in those with a firstborn boy compared with a firstborn girl.^32^ The study also revealed that birth of a girl was a significantly more common outcome of a live birth after a secondary RM, and that the maternal carriage of male‐specific H‐Y‐restricting HLA class II alleles was associated with the reduced birth rate of boys.

The major limitation was that there was no distinction among different etiologies for RPL, nor whether interventions were performed. The prevalence of early miscarriage was only .36% because many of the participants were recruited after 10 weeks’ gestation. Thus, it was one of the limitations that the early miscarriage and pregnancy loss results might not be reliable.

The present study represents the largest nationwide birth cohort study in Japan. The results are reliable because pregnancy and delivery information was drawn from medical records by doctors and research coordinators. The comparisons between complete‐case analyses and MIA allowed for a relevant sensitivity analysis to quantify the risk of a response bias. MIA allowed for a reliable estimation of the results and helped to minimize the risk of bias. ORs tended to be lower after the adjustment with MIA when they were compared using Model II and III. Many of covariates included in propensity score increased the rate of missing data, which might lead to over‐adjustment in Model II. Thus, MIA might insure the stability of the results in Model III, compared with those in Model II. On the other hand, there were no remarkable differences were observed in the results of Model III and Model IV, suggesting few effects from covariates related to the gynecological history. We, therefore, assumed that the results of Model III as the most important and reliable estimation minimizing the risk of bias.

This information, especially the finding that there was no increased risk of a live birth with a congenital anomaly or aneuploidy in women with a history of RPL as compared to women with no history of pregnancy loss, could encourage women who might hesitate to attempt a subsequent pregnancy. Many patients with RPL are afraid that their baby will have an anomaly because an abnormal embryonic karyotype is the most common cause of RPL.

## CONFLICT OF INTEREST

The authors declare that they have no conflict of interest.

This study was funded by the Ministry of the Environment, Government of Japan.

M.S‐O. received grants from the Japanese Ministry of Education, Culture, Sports, Science and Technology for conducting other studies on the topic of RPL in the different patient population, and payment for lectures from Kaken Pharmaceutical Co. Ltd., Kissei Pharmaceutical Co., Aska Pharmaceutical Co. Ltd., Sekisui Medical Co. Ltd., Siemens Japan. The remaining authors report no conflict of interest.

## AUTHOR CONTRIBUTIONS

The JECS group conducted the nationwide study project. MSO designed the present study, analyzed the data, and wrote the first draft of the manuscript. TE organized the study team and was responsible for obtaining and analyzing the data. MK, a member of the JECS Steering Committee, was responsible for data acquisition and supervision of the study. MK and TO took the initiative in the launch of the Aichi regional subcohort of JECS. NS TM, HK, and TK analyzed the data. YY, TO, MT, MM, and SS were responsible for data acquisition. All authors interpreted the data, contributed to the writing of the manuscript and revised it critically for important intellectual content.

## Data Availability

Data sharing is not permitted by the JECS due to a government policy restricting the deposition of data containing personal information. See the reference for more details.^21^
