# Supplementary material for: Adverse pregnancy and perinatal outcome in patients with recurrent pregnancy loss: Multiple imputation analyses with propensity score adjustment applied to a large‐scale birth cohort of the Japan Environment and Children’s Study
Source: Am J Reprod Immunol. 2018 Dec 13;81(1):e13072. doi: 10.1111/aji.13072 (PMC6646903; doi:10.1111/aji.13072)
Supplement: Supplementary file 1 [file AJI-81-na-s001.docx]

**TABLE 2** Obstetric outcome of women according to the number of previous pregnancy losses

| | Number of pregnancy losses | Prevalence %, (n) | | OR, 95%CI Model I | | OR, 95%CI Model II | | Pooled OR, 95%CI Multiple imputation model III | | Pooled OR, 95%CI Multiple imputation model Ⅳ | | Covariates included in pro |
|---|---|---|---|---|---|---|---|---|---|---|---|---|
| Late miscarriage and stillbirth> 20 weeks gestation | No | 0.7 | (534/71,708) | 1 | | 1 | | 1 | | 1 | | Age, BMI, Smoking, ART, Previous li Gastroesophageal reflux disease, Gastri |
| | One | 1.0 | (158/16,233) | **1.31** | **1.10 - 1.57** | **1.27** | **1.03 - 1.57** | 1.18 | 0.99 - 1.42 | 1.18 | 0.99 - 1.41 | |
| | Two | 1.2 | (42/3,564) | **1.59** | **1.16 - 2.18** | **1.49** | **1.03 - 2.15** | 1.34 | 0.97 - 1.84 | 1.33 | 0.97 - 1.82 | |
| | Three | 1.6 | (17/1,034) | **2.23** | **1.37 - 3.63** | **2.12** | **1.23 - 3.65** | **1.76** | **1.04 - 2.96** | **1.72** | **1.05 - 2.83** | |
| | Missing, % (n) | | | 3.8 | (3,673) | 9.7 | (9,332) | 2.8 | (2,646) | 2.8 | (2,646) | |
| Early miscarriage<12 weeks gestation | No | 0.3 | (238/71,412) | 1 | | 1 | | 1 | | 1 | | Age, Ulcerative colitis |
| | One | 0.5 | (76/16,151) | **1.41** | **1.09 - 1.83** | **2.09** | **1.34 - 3.23** | **1.38** | **1.05 - 1.81** | **1.38** | **1.05 - 1.81** | |
| | Two | 0.3 | (9/3,531) | 0.76 | 0.39 - 1.49 | 1.11 | 0.40 - 3.08 | 0.68 | 0.35 - 1.33 | 0.68 | 0.35 - 1.33 | |
| | Three | 0.7 | (7/1,024) | 2.06 | 0.97 - 4.38 | 2.63 | 0.81 - 8.50 | 1.89 | 0.89 - 4.02 | 1.89 | 0.89 - 4.02 | |
| | Missing, % (n) | | | 4.3 | (4,094) | 9.3 | (8,927) | 3.2 | (3,052) | 3.2 | (3,052) | |
| Pregnancy loss | No | 1.1 | (772/71,946) | 1 | | 1 | | 1 | | 1 | | Age, BMI, Smoking, ART, Previous li Adenomyosis |
| | One | 1.4 | (234/16,309) | **1.34** | **1.16 - 1.56** | **1.38** | **1.14 - 1.67** | **1.21** | **1.04 - 1.41** | **1.21** | **1.05 - 1.40** | |
| | Two | 1.4 | (51/3,573) | **1.34** | **1.00 - 1.78** | **1.43** | **1.01 - 2.02** | 1.09 | 0.82 - 1.46 | 1.08 | 0.81 - 1.44 | |
| | Three | 2.3 | (24/1,041) | **2.18** | **1.44 - 3.28** | **2.20** | **1.34 - 3.60** | **1.68** | **1.12 - 2.52** | **1.73** | **1.14 - 2.62** | |
| | Missing, % (n) | | | 3.5 | (3,343) | 9.6 | (9,238) | 2.4 | (2,282) | 2.4 | (2,282) | |
| Preterm birth<37 weeks gestation | No | 4.8 | (3,419/71,356) | 1 | | 1 | | 1 | | 1 | | Age, Marital status, BMI, Smoking, Ed income), ART, Atopic Dermatitis, Coll IDDM, NIDDM, Gestational diabetes, I Congenital heart disease, Depression, D disorders, Gastric ulcer, Chronic nephr Uterine fibroids, Adenomyosis, Conge |
| | One | 5.0 | (810/16,126) | 1.05 | 0.97 - 1.14 | 0.96 | 0.88 - 1.05 | 0.98 | 0.90 - 1.06 | 0.97 | 0.90 - 1.05 | |
| | Two | 5.8 | (206/3,532) | **1.23** | **1.07 - 1.42** | 1.01 | 0.85 - 1.19 | 1.09 | 0.94 - 1.27 | 1.07 | 0.93 - 1.24 | |
| | Three | 7.1 | (73/1,025) | **1.52** | **1.20 - 1.94** | 1.23 | 0.94 - 1.61 | **1.29** | **1.01 - 1.65** | 1.27 | 0.99 - 1.62 | |
| | Missing, % (n) | | | 4.3 | (4,173) | 17.5 | (16,866) | 3.3 | (3,164) | 3.3 | (3,164) | |
| Preterm birth <34 weeks gestation | No | 1.1 | (793/71,356) | 1 | | 1 | | 1 | | 1 | | Age, Marital status, BMI, Smoking, Ed IDDM, NIDDM, Gestational diabetes, I Gastric ulcer, Fatty liver, Chronic neph Uterine fibroids, Adenomyosis, PCOS |
| | One | 1.3 | (207/16,126) | 1.16 | 1.00 - 1.36 | 1.06 | 0.89 - 1.27 | 1.05 | 0.90 - 1.23 | 1.05 | 0.90 - 1.23 | |
| | Two | 1.6 | (57/3,532) | **1.46** | **1.11 - 1.91** | 1.12 | 0.81 - 1.56 | 1.22 | 0.93 - 1.61 | 1.21 | 0.92 - 1.60 | |
| | Three | 2.0 | (21/1,025) | **1.86** | **1.20 - 2.88** | 1.17 | 0.67 - 2.05 | 1.43 | 0.92 - 2.23 | 1.41 | 0.90 - 2.21 | |
| | Missing, % (n) | | | 4.3 | (4,173) | 11.8 | (11,313) | 3.3 | (3,164) | 3.3 | (3,164) | |
| Premature rupture of membrane | No | 9.3 | (6,622/71,392) | 1 | | 1 | | 1 | | 1 | | Age, Marital status, Smoking, Educatic ART, Previous live birth, SLE, IDDM, disease, Uterine fibroids, Adenomyosis |
| | One | 8.2 | (1,315/16,131) | **0.87** | **0.82 - 0.92** | 0.95 | 0.89 - 1.02 | 0.96 | 0.90 - 1.02 | 0.96 | 0.90 - 1.02 | |
| | Two | 8.2 | (291/3,533) | **0.88** | **0.78 - 0.99** | 1.06 | 0.93 - 1.21 | 1.03 | 0.91 - 1.17 | 1.03 | 0.91 - 1.17 | |
| | Three | 8.4 | (86/1,025) | 0.90 | 0.71 - 1.10 | 1.07 | 0.84 - 1.36 | 1.11 | 0.89 - 1.39 | 1.12 | 0.89 - 1.41 | |
| | Missing, % (n) | | | 4.3 | (4,131) | 17.5 | (16,828) | 3.2 | (3,120) | 3.2 | (3,120) | |
| Placenta praevia | No | 0.6 | (438/71,392) | 1 | | 1 | | 1 | | 1 | | Age, Household income, ART, Gestatic Endometriosis, Uterine fibroids, Ovari |
| | One | 0.8 | (137/16,131) | **1.39** | **1.14 - 1.68** | **1.25** | **1.02 - 1.53** | **1.26** | **1.04 - 1.53** | **1.24** | **1.02 - 1.51** | |
| | Two | 1.1 | (40/3,533) | **1.86** | **1.34 - 2.57** | **1.44** | **1.01 - 2.06** | **1.56** | **1.12 - 2.17** | **1.50** | **1.08 - 2.09** | |
| | Three | 1.1 | (11/1,025) | 1.76 | 0.96 - 3.21 | 1.30 | 0.69 - 2.46 | 1.34 | 0.73 - 2.46 | 1.27 | 0.70 - 2.34 | |
| | Missing, % (n) | | | 4.3 | (4,131) | 16.4 | (15,814) | 3.2 | (3,120) | 3.2 | (3,120) | |

## **TABLE 2** Continued

| | Number of pregnancy losses | Prevalence %, | (n) | OR, 95%CI Model I | | Model II | | Pooled OR, 95%CI Multiple imputation model III | | Multiple imputation model Ⅳ | | Covariates include |
|---|---|---|---|---|---|---|---|---|---|---|---|---|
| Abruptio placentae | No | 0.4 | (292/71,392) | 1 | | 1 | | 1 | | 1 | | Age, Smoking, ART, Adenomy |
| | One | 0.5 | (84/16,131) | 1.28 | 1.00 - 1.63 | 1.15 | 0.89 - 1.49 | 1.21 | 0.95 - 1.55 | 1.19 | 0.93 - 1.53 | |
| | Two | 0.5 | (18/3,533) | 1.25 | 0.77 - 2.01 | 1.05 | 0.63 - 1.75 | 1.13 | 0.70 - 1.83 | 1.13 | 0.70 - 1.83 | |
| | Three | 0.7 | (7/1,025) | 1.67 | 0.79 - 3.55 | 1.30 | 0.57 - 2.93 | 1.47 | 0.69 - 3.14 | 1.41 | 0.66 - 3.00 | |
| | Missing, % (n) | | | 4.3 | (4,131) | 9.8 | (9,419) | 3.2 | (3,120) | 3.2 | (3,120) | |
| Placental adhesion | No | 0.2 | (150/71,392) | 1 | | 1 | | 1 | | 1 | | Age, ART, Hyperthyroidism, E Congenital uterine anomalies, P |
| | One | 0.3 | (45/16,131) | 1.33 | 0.95 - 1.86 | 1.20 | 0.84 - 1.70 | 1.23 | 0.88 - 1.72 | 1.17 | 0.84 - 1.64 | |
| | Two | 0.4 | (15/3,533) | **2.03** | **1.19 - 3.45** | **1.83** | **1.07 - 3.14** | **1.74** | **1.01 - 2.98** | 1.64 | 0.95 - 2.82 | |
| | Three | 0.7 | (7/1,025) | **3.27** | **1.53 - 6.99** | **2.69** | **1.24 - 5.82** | **2.53** | **1.17 - 5.47** | **2.33** | **1.08 - 5.04** | |
| | Missing, % (n) | | | 4.3 | (4,131) | 9.1 | (8,767) | 3.2 | (3,120) | 3.2 | (3,120) | |
| Hypertensive disorders of pregnancy (mild) | No | 2.3 | (1659/71,392) | 1 | | 1 | | 1 | | 1 | | Age, BMI, Smoking, Education Previous live birth, Bronchial as Gestational diabetes, Hyperthyr pressure, Fatty liver, Chronic ne Adenomyosis |
| | One | 2.2 | (354/16,131) | 0.94 | 0.84 - 1.06 | 0.95 | 0.84 - 1.07 | 0.94 | 0.84 - 1.06 | 0.95 | 0.84 - 1.06 | |
| | Two | 2.5 | (90/3,533) | 1.10 | 0.89 - 1.36 | 1.09 | 0.86 - 1.37 | 1.10 | 0.88 - 1.36 | 1.10 | 0.88 - 1.36 | |
| | Three | 4.3 | (44/1,025) | **1.89** | **1.39 - 2.56** | **2.02** | **1.46 - 2.78** | **1.87** | **1.37 - 2.55** | **1.88** | **1.38 - 2.56** | |
| | Missing, % (n) | | | 4.3 | (4,131) | 17.3 | (16,626) | 3.2 | (3,120) | 3.2 | (3,120) | |
| Hypertensive disorders of pregnancy (severe) | No | 1.0 | (695/71,392) | 1 | | 1 | | 1 | | 1 | | Age, BMI, ART, Previous live b blood pressure, High cholestero Uterine fibroids, Congenital ute |
| | One | 1.1 | (171/16,131) | 1.09 | 0.92 - 1.29 | 0.96 | 0.80 - 1.14 | 1.04 | 0.88 - 1.23 | 1.03 | 0.87 - 1.22 | |
| | Two | 0.9 | (32/3,533) | 0.93 | 0.65 - 1.33 | 0.75 | 0.53 - 1.08 | 0.84 | 0.59 - 1.21 | 0.85 | 0.60 - 1.22 | |
| | Three | 1.8 | (18/1,025) | **1.82** | **1.13 - 2.92** | **1.29** | **0.80 - 2.08** | 1.60 | 0.99 - 2.57 | 1.63 | 1.00 - 2.64 | |
| | Missing, % (n) | | | 4.3 | (4,131) | 9.2 | (8,892) | 3.2 | (3,120) | 3.2 | (3,120) | |
| Uterine infection | No | 0.7 | (535/71,980) | 1 | | 1 | | 1 | | 1 | | Marital status, BMI, Education, Endometriosis |
| | One | 0.7 | (114/16,312) | 0.94 | 0.77 - 1.15 | 1.06 | 0.85 - 1.33 | 1.11 | 0.90 - 1.36 | 1.11 | 0.90 - 1.36 | |
| | Two | 0.8 | (29/3,574) | 1.09 | 0.75 - 1.59 | 1.26 | 0.83 - 1.93 | 1.42 | 0.97 - 2.07 | 1.40 | 0.96 - 2.04 | |
| | Three | 1.1 | (11/1,041) | 1.43 | 0.78 - 2.60 | 1.62 | 0.80 - 3.28 | **1.94** | **1.06 - 3.55** | **1.96** | **1.07 - 3.59** | |
| | Missing, % (n) | | | 3.4 | (3,305) | 7.7 | (7,405) | 2.3 | (2,242) | 2.3 | (2,242) | |
| Caesarean section | No | 18.2 | (12,978/71,126) | 1 | | 1 | | 1 | | 1 | | Age, Marital status, BMI, Smok income), ART, Atopic Dermatit *disease*, SLE, RA, IDDM, NIDD Hypothyroidism, High blood pr disease, *Depression*, Anxiety dis *disease*, *Gastritis*, Gastric ulcer, Chronic nephritis, Endometriosi Adenomyosis, Congenital uterin |
| | One | 20.4 | (3,278/16,081) | **1.15** | **1.10 - 1.20** | 1.04 | 0.99 - 1.09 | 1.03 | 0.99 - 1.08 | 1.03 | 0.98 - 1.07 | |
| | Two | 23.8 | (840/3,524) | **1.40** | **1.30 - 1.52** | **1.13** | **1.04 - 1.24** | **1.17** | **1.08 - 1.27** | **1.15** | **1.06 - 1.25** | |
| | Three | 27.2 | (277/1,019) | **1.67** | **1.46 - 1.92** | **1.31** | **1.13 - 1.52** | **1.28** | **1.11 - 1.47** | **1.25** | **1.08 - 1.44** | |
| | Missing, % (n) | | | 4.6 | (4,462) | 17.8 | (17,089) | 3.6 | (3,460) | 3.6 | (3,460) | |

Model I  Unadjusted model
Model II  Adjusted model using propensity scores based on complete case analysis
Model III  Adjusted model using propensity scores based on multiple imputation model
Model IV  Covariates about gynecological history (underlined covariates) were added to the model III
Values in boldface indicated a statistical significance **level of 0.05**

**TABLE 3** Perinatal outcome of children according to the number of previous pregnancy losses

| | Number of pregnancy losses | Prevalence %, (n) | | OR, 95%CI | | | | Pooled OR, 95%CI | | | | Covariates included |
|---|---|---|---|---|---|---|---|---|---|---|---|---|
| | | | | Model I | | Model II | | Multiple imputation model III | | Multiple imputation model Ⅳ | | |
| SFD | No | 10.3 | (7,370/71,271) | 1 | | 1 | | 1 | | 1 | | Age, Marital status, BMI, Smok<br>disease, SLE, IDDM, Anemia, H<br>heart disease, Adenomyosis, PC |
| | One | 9.2 | (1,486/16,106) | **0.88** | **0.83 - 0.93** | 0.97 | 0.91 - 1.03 | 0.96 | 0.91 - 1.02 | 0.96 | 0.91 - 1.02 | |
| | Two | 8.7 | (306/3,528) | **0.82** | **0.73 - 0.93** | 0.95 | 0.84 - 1.07 | 0.96 | 0.85 - 1.08 | 0.96 | 0.85 - 1.09 | |
| | Three | 8.6 | (88/1,025) | 0.81 | 0.65 - 1.01 | 0.99 | 0.78 - 1.24 | 0.98 | 0.79 - 1.23 | 1.01 | 0.81 - 1.26 | |
| | Missing, % (n) | | | 4.5 | (4,282) | 10.5 | (10,124) | 3.4 | (3,273) | 3.4 | (3,273) | |
| IUFD | No | 0.4 | (294/71,980) | 1 | | 1 | | 1 | | 1 | | Age, BMI, Adenomyosis |
| | One | 0.5 | (80/16,312) | 1.20 | 0.94 - 1.54 | 1.11 | 0.86 - 1.43 | 1.10 | 0.86 - 1.42 | 1.11 | 0.86 - 1.42 | |
| | Two | 0.4 | (16/3,574) | 1.10 | 0.66 - 1.82 | 0.95 | 0.57 - 1.58 | 0.94 | 0.56 - 1.56 | 0.92 | 0.55 - 1.53 | |
| | Three | 0.8 | (8/1,041) | 1.89 | 0.93 - 3.82 | 1.53 | 0.75 - 3.12 | 1.55 | 0.76 - 3.16 | 1.53 | 0.76 - 3.11 | |
| | Missing, % (n) | | | 3.4 | (3,305) | 7.2 | (6,965) | 2.3 | (2,242) | 2.3 | (2,242) | |
| Low birth weight<2500g | No | 8.5 | (6,051/71,300) | 1 | | 1 | | 1 | | 1 | | Age, Marital status, BMI, Smok<br>live birth, Collagen disease, SLE<br>pressure, Congenital heart disea<br>disorders, Chronic nephritis, Ne<br>Endometriosis, Uterine fibroids,<br>uterine anomalies |
| | One | 7.9 | (1,274/16,115) | **0.93** | **0.87 - 0.99** | **0.92** | **0.86 - 0.98** | **0.93** | **0.87 - 0.99** | **0.92** | **0.86 - 0.98** | |
| | Two | 9.1 | (321/3,529) | 1.08 | 0.96 - 1.21 | 1.04 | 0.91 - 1.18 | 1.09 | 0.97 - 1.23 | 1.06 | 0.95 - 1.20 | |
| | Three | 10.0 | (102/1,024) | 1.19 | 0.97 - 1.47 | 1.18 | 0.95 - 1.47 | 1.20 | 0.98 - 1.47 | 1.17 | 0.95 - 1.45 | |
| | Missing, % (n) | | | 4.4 | (4,244) | 11.9 | (11,438) | 3.4 | (3,236) | 3.4 | (3,236) | |
| Male | No | 51.3 | (36,804/71,700) | 1 | | 1 | | 1 | | 1 | | Gestational diabetes, Dysautono |
| | One | 51.8 | (8,402/16,219) | **1.02** | **0.98 - 1.05** | 1.02 | 0.98 - 1.05 | 1.02 | 0.99 - 1.06 | 1.02 | 0.99 - 1.06 | |
| | Two | 51.8 | (1,844/3,557) | **1.02** | **0.95 - 1.09** | 1.02 | 0.95 - 1.09 | 1.02 | 0.95 - 1.09 | 1.02 | 0.95 - 1.09 | |
| | Three | 47.7 | (493/1,034) | **0.86** | **0.76 - 0.98** | **0.87** | **0.77 - 0.98** | **0.86** | **0.76 - 0.98** | **0.86** | **0.76 - 0.98** | |
| | Missing, % (n) | | | 3.8 | (3,702) | 5.2 | (4,987) | 2.8 | (2,676) | 2.8 | (2,676) | |
| Apgar score <7 at 5 minute | No | 0.6 | (419/67,588) | 1 | | 1 | | 1 | | 1 | | Age, BMI, Smoking, ART, Prev<br>Dermatitis, IDDM, NIDDM, Hig<br>disease, Uterine fibroids, Aden |
| | One | 0.8 | (117/15,337) | **1.23** | **1.00 - 1.51** | **1.24** | **1.00 - 1.54** | 1.20 | 0.98 - 1.49 | 1.19 | 0.97 - 1.47 | |
| | Two | 0.7 | (22/3,364) | 1.06 | 0.69 - 1.62 | 1.05 | 0.67 - 1.64 | 1.00 | 0.64 - 1.54 | 1.00 | 0.65 - 1.55 | |
| | Three | 0.5 | (5/978) | 0.82 | 0.34 - 1.99 | 0.51 | 0.16 - 1.59 | 0.76 | 0.31 - 1.84 | 0.74 | 0.31 - 1.81 | |
| | Missing, % (n) | | | 9.3 | (8,945) | 14.7 | (14,184) | 8.3 | (8,015) | 8.3 | (8,015) | |
| pH<7.1 | No | 1.2 | (697/59,523) | 1 | | 1 | | 1 | | 1 | | ART, Previous live birth, NIDD |
| | One | 1.2 | (166/13,545) | 1.05 | 0.88 - 1.24 | 1.16 | 0.98 - 1.38 | 1.16 | 0.98 - 1.39 | 1.16 | 0.98 - 1.39 | |
| | Two | 1.2 | (36/2,947) | 1.04 | 0.75 - 1.46 | 1.22 | 0.86 - 1.72 | 1.22 | 0.87 - 1.71 | 1.22 | 0.87 - 1.71 | |
| | Three | 1.1 | (10/871) | 0.98 | 0.52 - 1.84 | 1.10 | 0.57 - 2.13 | 1.20 | 0.64 - 2.27 | 1.20 | 0.64 - 2.27 | |
| | Missing, % (n) | | | 20.1 | (19,326) | 21.1 | (20,337) | 19.3 | (18,587) | 19.3 | (18,587) | |
| Congenital anomaly | No | 12.2 | (8,607/70,321) | 1 | | 1 | | 1 | | 1 | | Age, BMI, Education, (Househo<br>disease, IDDM, Gestational diab<br>Hypothyroidism, High blood pr<br>Depression, Gastritis, Irritable b<br>Adenomyosis |
| | One | 12.6 | (2,004/15,899) | 1.03 | 0.98 - 1.09 | 1.01 | 0.95 - 1.06 | 1.01 | 0.96 - 1.06 | 1.01 | 0.96 - 1.06 | |
| | Two | 13.2 | (461/3,492) | 1.09 | 0.99 - 1.21 | 1.01 | 0.91 - 1.13 | 1.04 | 0.94 - 1.15 | 1.04 | 0.94 - 1.15 | |
| | Three | 12.7 | (129/1,015) | 1.04 | 0.87 - 1.26 | 0.97 | 0.80 - 1.18 | 0.96 | 0.80 - 1.16 | 0.96 | 0.80 - 1.16 | |
| | Missing, % (n) | | | 5.7 | (5,485) | 17.8 | (17,142) | 4.7 | (4,505) | 4.7 | (4,505) | |
| Aneuploidy | No | 0.2 | (140/71,424) | 1 | | 1 | | 1 | | 1 | | Age, Smoking, Previous live bi<br>Depression, Uterine fibroids, Ad |
| | One | 0.2 | (38/16,136) | 1.20 | 0.84 - 1.72 | 0.94 | 0.65 - 1.37 | 0.99 | 0.69 - 1.42 | 0.97 | 0.67 - 1.39 | |
| | Two | 0.2 | (8/3,533) | 1.16 | 0.57 - 2.36 | 0.73 | 0.34 - 1.58 | 0.80 | 0.39 - 1.64 | 0.82 | 0.40 - 1.68 | |
| | Three | 0.2 | (2/1,025) | 1.00 | 0.25 - 4.03 | 0.63 | 0.16 - 2.58 | 0.61 | 0.15 - 2.46 | 0.59 | 0.15 - 2.31 | |

**TABLE S1** Obstetric outcome of women according to the number of previous artificial abortions

| | Number of induced abortions | Prevalence %, (n) | | OR, 95%CI Model I | | Model II | | Pooled OR, 95%CI Multiple imputation model Ⅲ | | Multiple imputation model Ⅳ | | Included cc |
|---|---|---|---|---|---|---|---|---|---|---|---|---|
| Placental adhesion | No | 0.2 | (167/75,441) | 1 | | 1 | | 1 | | 1 | | Age, ART, Hy |
| | One | 0.3 | (35/12,545) | 1.26 | 0.88 - 1.82 | 1.31 | 0.90 - 1.92 | 1.33 | 0.93 - 1.92 | 1.33 | 0.92 - 1.91 | Adenomyosis |
| | Two | 0.3 | (9/3,329) | 1.22 | 0.62 - 2.39 | 1.29 | 0.63 - 2.64 | 1.34 | 0.68 - 2.62 | 1.41 | 0.72 - 2.76 | PCOS |
| | Three | 0.5 | (6/1,116) | **2.44** | **1.08 - 5.51** | 1.93 | 0.71 - 5.23 | **2.67** | **1.19 - 5.99** | **2.72** | **1.20 - 6.16** | |
| | Missing, % (n) | | | 3.9 | (3,781) | 8.8 | (8,426) | 3.2 | (3,120) | 3.2 | (3,120) | |
| Uterine infection | No | 0.7 | (545/76,121) | 1 | | 1 | | 1 | | 1 | | Marital status |
| | One | 0.9 | (108/12,650) | 1.194 | 0.97 - 1.47 | 1.27 | 1.02 - 1.59 | **1.31** | **1.06 - 1.62** | **1.31** | **1.06 - 1.61** | live birth, Enc |
| | Two | 0.7 | (25/3,360) | 1.04 | 0.70 - 1.55 | 1.10 | 0.70 - 1.72 | 1.21 | 0.80 - 1.81 | 1.23 | 0.82 - 1.85 | |
| | Three | 1.2 | (13/1,125) | 1.621 | 0.93 - 2.82 | 1.43 | 0.71 - 2.90 | **2.01** | **1.15 - 3.52** | **2.02** | **1.16 - 3.53** | |
| | Missing, % (n) | | | 3.1 | (2,956) | 7.4 | (7,077) | 2.3 | (2,242) | 2.3 | (2,242) | |
