# Supplementary material for: Adverse pregnancy and perinatal outcome in patients with recurrent pregnancy loss: Multiple imputation analyses with propensity score adjustment applied to a large‐scale birth cohort of the Japan Environment and Children’s Study
Source: Am J Reprod Immunol. 2018 Dec 13;81(1):e13072. doi: 10.1111/aji.13072 (PMC6646903; doi:10.1111/aji.13072)
Supplement: Supplementary file 2 [file AJI-81-na-s002.docx]

TABLE S2 Demographic characteristics of women according to the number of previous pregnancy losses (n=100,2[illegible]

Variables	No miscarriage (n=77,434)		One (n=17,733)		Two (n=3,907)		Three or more (n=1,131)	
Age (years), % (n)								
<20	1.4	(1,048)	0.3	(58)	0.2	(8)	0.3	(3[illegible]
20-29	43.1	(32,089)	30.2	(5,160)	22.0	(830)	16.2	(17[illegible]
30-39	52.8	(39,322)	64.2	(10,960)	68.1	(2,567)	67.4	(73[illegible]
>40	2.8	(2,053)	5.3	(902)	9.7	(367)	16.1	(17[illegible]
Missing, % (n)	3.8	(2,922)	3.7	(653)	3.5	(135)	4.0	(4[illegible]
BMI, % (n)								
<18.5	16.7	(12,902)	14.8	(2,616)	14.1	(551)	14.5	(16[illegible]
18.5-25.0	72.8	(56,262)	73.8	(13,062)	73.1	(2,852)	72.7	(82[illegible]
≥25.0	10.4	(8,068)	11.4	(2,012)	12.7	(496)	12.7	(14[illegible]
Missing, % (n)	0.3	(202)	0.2	(43)	0.2	(8)	0.1	([illegible]
Marital status, % (n)								
Married	95.2	(71,449)	97.2	(16,829)	97.7	(3,726)	96.7	(1,07[illegible]
Single	4.1	(3,086)	1.7	(289)	0.9	(34)	0.8	([illegible]
Divorced	0.7	(531)	1.1	(194)	1.4	(55)	2.5	(2[illegible]
Missing, % (n)	3.1	(2,368)	2.4	(421)	2.4	(92)	2.1	(2[illegible]
IVF-ET, % (n)								
Carried out	2.6	(2,011)	4.1	(724)	5.1	(198)	9.0	(10[illegible]
Missing, % (n)	0.0	(20)	0.0	(4)	0.0	(1)	0.0	([illegible]
Previous live birth, % (n)								
Yes	54.6	(42,259)	70.4	(12,440)	77.6	(3,027)	79.8	(90[illegible]
Missing, % (n)	0.0	(21)	0.3	(50)	0.2	(8)	0.2	([illegible]
Smoking, % (n)								
Never smoked	59.4	(44,435)	55.6	(9,588)	53.0	(2,019)	51.6	(56[illegible]
Quit smoking before pregnancy	22.6	(16,930)	27.1	(4,672)	26.9	(1,025)	30.4	(33[illegible]
Quit smoking during early pregnancy	13.5	(10,085)	12.0	(2,063)	12.9	(493)	11.0	(12[illegible]
Current smoker	4.5	(3,392)	5.4	(928)	7.1	(272)	7.0	(7[illegible]
Missing, % (n)	3.3	(2,592)	2.7	(482)	2.5	(98)	2.6	(2[illegible]
Educational background (years), % (n)								
Junior high/ High school	35.9	(26,499)	36.6	(6,218)	39.8	(1,481)	42.2	(45[illegible]
College/Junior college/Technology college	41.8	(30,847)	43.2	(7,330)	41.8	(1,555)	41.6	(44[illegible]
University	20.8	(15,335)	18.8	(3,193)	17.4	(646)	15.3	(16[illegible]
Graduate school	1.5	(1,135)	1.4	(231)	1.0	(38)	0.9	(1[illegible]
Missing, % (n)	4.7	(3,618)	4.3	(761)	4.8	(187)	5.0	(5[illegible]
Income (JPY), % (n)								
<200	5.8	(3,958)	5.3	(846)	5.5	(191)	6.6	(6[illegible]
200-<400	35.0	(24,109)	33.4	(5,322)	32.1	(1,119)	31.0	(31[illegible]
400-<600	32.8	(22,601)	33.9	(5,412)	32.7	(1,140)	33.5	(34[illegible]
600-<800	15.7	(10,813)	16.4	(2,610)	18.0	(626)	16.3	(16[illegible]
800-<1,000	6.5	(4,464)	6.5	(1,038)	6.9	(240)	8.2	(8[illegible]
≥1,000	4.2	(2,879)	4.6	(728)	4.8	(168)	4.4	(4[illegible]
