# Supplementary material for: Adverse pregnancy and perinatal outcome in patients with recurrent pregnancy loss: Multiple imputation analyses with propensity score adjustment applied to a large‐scale birth cohort of the Japan Environment and Children’s Study
Source: Am J Reprod Immunol. 2018 Dec 13;81(1):e13072. doi: 10.1111/aji.13072 (PMC6646903; doi:10.1111/aji.13072)
Supplement: Supplementary file 3 [file AJI-81-na-s003.docx]

TABLE S3 Obstetric outcome of women according to the number of previous pregnancy losses

	Number of pregnancy losses	Prevalence %, (n)		OR, 95%CI Model I		OR, 95%CI Model II		Pooled OR, 95%CI Multiple imputation model III		Pooled OR, 95%CI Multiple imputation model IV		Covariates included in pro
Late miscarriage and stillbirth>20 weeks gestation	No	0.7	(562/75,688)	1		1		1		1		Age, BMI, Smoking, ART, Previous liv Gastroesophageal reflux disease, Gastri
	One	1.0	(170/17,404)	**1.32**	**1.11 - 1.57**	**1.28**	**1.04 - 1.57**	1.18	0.99 - 1.40	1.18	0.99 - 1.40	
	Two	1.3	(50/3,834)	**1.77**	**1.32 - 2.36**	**1.68**	**1.20 - 2.35**	**1.48**	**1.10 - 1.99**	**1.49**	**1.11 - 2.00**	
	Three	1.6	(18/1,099)	**2.23**	**1.39 - 3.57**	**2.12**	**1.25 - 3.58**	**1.71**	**1.06 - 2.75**	**1.79**	**1.12 - 2.85**	
	Missing, % (n)			3.7	(3,773)	9.6	(9,743)	2.7	(2,719)	2.7	(2,719)	
Early miscarriage<12 weeks gestation	No	0.3	(261/75,387)	1		1		1		1		Age, Previous live birth, Ulcerative col
	One	0.5	(84/17,318)	**1.40**	**1.10 - 1.80**	**1.77**	**1.16 - 2.71**	1.17	0.91 - 1.50	1.20	0.94 - 1.53	
	Two	0.3	(10/3,794)	0.76	0.40 - 1.43	0.90	0.33 - 2.49	0.59	0.32 - 1.10	0.61	0.32 - 1.16	
	Three	0.6	(7/1,088)	1.86	0.88 - 3.96	2.17	0.67 - 7.01	1.34	0.62 - 2.93	1.42	0.59 - 3.40	
	Missing, % (n)			4.1	(4,211)	9.2	(9,395)	3.1	(3,139)	3.1	(3,139)	
Pregnancy loss	No	1.1	(823/75,949)	1		1		1		1		Age, BMI, Smoking, ART, Previous liv Adenomyosis
	One	1.5	(254/17,488)	**1.35**	**1.17 - 1.55**	**1.35**	**1.12 - 1.62**	**1.21**	**1.04 - 1.39**	**1.18**	**1.02 - 1.36**	
	Two	1.6	(60/3,844)	**1.45**	**1.11 - 1.89**	**1.56**	**1.13 - 2.14**	1.21	0.91 - 1.61	1.17	0.89 - 1.53	
	Three	2.3	(25/1,106)	**2.11**	**1.41 - 3.16**	**2.13**	**1.32 - 3.45**	**1.57**	**1.05 - 2.37**	**1.63**	**1.09 - 2.44**	
	Missing, % (n)			3.4	(3,411)	9.5	(9,639)	2.3	(2,319)	2.3	(2,319)	
Preterm birth<37 weeks gestation	No	4.8	(3,605/75,316)	1		1		1		1		Age, Marital status, BMI, Smoking, Ed income), ART, Atopic Dermatitis, Colla IDDM, NIDDM, Gestational diabetes, I Congenital heart disease, Depression, D disorders, Gastric ulcer, Chronic nephri Uterine fibroids, Adenomyosis, Conger
	One	5.1	(875/17,289)	1.06	0.98 - 1.14	0.98	0.90 - 1.06	0.99	0.92 - 1.07	0.98	0.91 - 1.06	
	Two	5.9	(222/3,794)	**1.24**	**1.08 - 1.42**	1.04	0.88 - 1.21	1.10	0.95 - 1.26	1.09	0.94 - 1.25	
	Three	7.5	(82/1,089)	**1.62**	**1.29 - 2.03**	**1.34**	**1.04 - 1.72**	**1.36**	**1.08 - 1.71**	**1.35**	**1.07 - 1.70**	
	Missing, % (n)			4.2	(4,310)	17.3	(17,644)	3.2	(3,275)	3.2	(3,275)	
Preterm birth <34 weeks gestation	No	1.1	(832/75,316)	1		1		1		1		Age, Marital status, BMI, Smoking, Ed live birth, SLE, RA, IDDM, NIDDM, G blood pressure, Gastric ulcer, Chronic r Uterine fibroids, Adenomyosis, PCOS
	One	1.3	(225/17,289)	**1.18**	**1.02 - 1.37**	1.08	0.91 - 1.29	1.08	0.93 - 1.25	1.07	0.92 - 1.25	
	Two	1.5	(58/3,794)	**1.39**	**1.06 - 1.82**	1.08	0.78 - 1.49	1.17	0.89 - 1.53	1.18	0.89 - 1.56	
	Three	1.9	(21/1,089)	**1.76**	**1.14 - 2.73**	1.12	0.64 - 1.95	1.38	0.89 - 2.15	1.38	0.89 - 2.14	
	Missing, % (n)			4.2	(4,310)	11.6	(11,833)	3.2	(3,275)	3.2	(3,275)	
Premature rupture of membrane	No	9.0	(6,805/75,352)	1		1		1		1		Age, Marital status, Smoking, Educatio ART, Previous live birth, SLE, IDDM, disease, Uterine fibroids, PCOS
	One	8.0	(1,391/17,294)	**0.88**	**0.83 - 0.94**	0.96	0.90 - 1.03	0.97	0.91 - 1.03	0.96	0.91 - 1.03	
	Two	8.0	(303/3,795)	**0.87**	**0.78 - 0.99**	1.05	0.93 - 1.20	1.02	0.91 - 1.16	1.02	0.91 - 1.15	
	Three	8.4	(91/1,089)	0.92	0.74 - 1.14	1.08	0.85 - 1.37	1.14	0.92 - 1.41	1.15	0.92 - 1.43	
	Missing, % (n)			4.2	(4,268)	17.3	(17,604)	3.2	(3,231)	3.2	(3,231)	
Placenta praevia	No	0.6	(453/75,352)	1		1		1		1		Age, (Household income), ART, Gestat Endometriosis, Uterine fibroids, Ovaria
	One	0.9	(148/17,294)	**1.43**	**1.19 - 1.72**	**1.27**	**1.04 - 1.55**	**1.30**	**1.08 - 1.57**	**1.29**	**1.07 - 1.55**	
	Two	1.1	(40/3,795)	**1.76**	**1.27 - 2.44**	1.37	0.96 - 1.96	**1.48**	**1.06 - 2.05**	**1.46**	**1.06 - 2.03**	
	Three	1.1	(12/1,089)	**1.84**	**1.04 - 3.28**	1.38	0.75 - 2.53	1.38	0.77 - 2.48	1.35	0.76 - 2.42	
	Missing, % (n)			4.2	(4,268)	16.2	(16,542)	3.2	(3,231)	3.2	(3,231)	
Oligohydramnios	No	1.3	(965/75,352)	1		1		1		1		Marital status, BMI, (Household incom birth, Gestational diabetes, Depression
	One	1.2	(214/17,294)	0.97	0.83 - 1.12	1.06	0.90 - 1.25	1.08	0.93 - 1.26	1.08	0.93 - 1.26	
	Two	1.5	(56/3,795)	1.16	0.88 - 1.52	**1.40**	**1.05 - 1.87**	**1.37**	**1.04 - 1.79**	**1.37**	**1.04 - 1.79**	

TABLE S3 Continued

	Number of pregnancy losses	Prevalence, %	(n)	OR, 95%CI Model I		OR, 95%CI Model II		Pooled OR, 95%CI Multiple imputation model III		Pooled OR, 95%CI Multiple imputation model IV		Covariates included
Abruptio placentae	No	0.4	(308/75,352)	1		1		1		1		Age, Smoking, ART
	One	0.5	(90/17,294)	**1.28**	**1.01 - 1.61**	1.15	0.90 - 1.48	1.22	0.96 - 1.55	1.22	0.96 - 1.55	
	Two	0.5	(19/3,795)	1.23	0.77 - 1.95	1.05	0.64 - 1.72	1.12	0.70 - 1.80	1.12	0.70 - 1.80	
	Three	0.9	(10/1,089)	**2.26**	**1.20 - 4.25**	1.86	0.95 - 3.64	**1.99**	**1.05 - 3.79**	**1.99**	**1.05 - 3.79**	
	Missing, % (n)			4.2	(4,268)	9.7	(9,848)	3.2	(3,231)	3.2	(3,231)	
Placental adhesion	No	0.2	(158/75,352)	1		1		1		1		Age, ART, Hyperthyroidism, H
	One	0.3	(49/17,294)	1.35	0.98 - 1.86	1.21	0.87 - 1.70	1.22	0.89 - 1.69	1.20	0.87 - 1.65	Adenomyosis, Congenital uteri
	Two	0.4	(15/3,795)	**1.89**	**1.11 - 3.21**	1.68	0.98 - 2.88	1.53	0.90 - 2.61	1.49	0.87 - 2.55	PCOS
	Three	0.6	(7/1,089)	**3.08**	**1.44 - 6.58**	**2.47**	**1.14 - 5.33**	**2.30**	**1.07 - 4.95**	**2.16**	**1.000 - 4.66**	
	Missing, % (n)			4.2	(4,268)	9.0	(9,160)	3.2	(3,231)	3.2	(3,231)	
Hypertensive disorders of pregnancy (mild)	No	2.3	(1,710/75,352)	1		1		1		1		Age, BMI, Smoking, Education
	One	2.2	(376/17,294)	0.96	0.86 - 1.07	0.96	0.85 - 1.09	0.96	0.85 - 1.07	0.95	0.85 - 1.07	Previous live birth, IDDM, NIC
	Two	2.5	(93/3,795)	1.08	0.88 - 1.34	1.06	0.84 - 1.33	1.07	0.87 - 1.33	1.06	0.86 - 1.31	Hyperthyroidism, Anemia, Hig
	Three	4.1	(45/1,089)	**1.86**	**1.37 - 2.51**	**1.95**	**1.42 - 2.68**	**1.82**	**1.34 - 2.47**	**1.83**	**1.35 - 2.48**	disorders, Fatty liver, Chronic n
	Missing, % (n)			4.2	(4,268)	17.1	(17,401)	3.2	(3,231)	3.2	(3,231)	Adenomyosis
Hypertensive disorders of pregnancy (severe)	No	0.9	(712/75,352)	1		1		1		1		Age, BMI, ART, Nulliparous, N
	One	1.0	(177/17,294)	1.08	0.92 - 1.28	1.03	0.87 - 1.22	1.03	0.87 - 1.22	1.03	0.87 - 1.22	Anemia, High blood pressure, I
	Two	0.8	(32/3,795)	0.89	0.63 - 1.27	0.86	0.60 - 1.24	0.83	0.58 - 1.19	0.82	0.57 - 1.17	Chronic nephritis, Endometrios
	Three	1.7	(19/1,089)	**1.86**	**1.18 - 2.95**	**1.76**	**1.10 - 2.79**	**1.64**	**1.03 - 2.60**	**1.67**	**1.05 - 2.66**	PCOS
	Missing, % (n)			4.2	(4,268)	9.2	(9,364)	3.2	(3,231)	3.2	(3,231)	
Uterine infection	No	0.7	(552/75,983)	1		1		1		1		Marital status, Education, ART,
	One	0.7	(115/17,491)	0.90	0.74 - 1.11	1.02	0.82 - 1.27	1.07	0.87 - 1.31	1.06	0.87 - 1.30	Endometriosis
	Two	0.8	(31/3,845)	1.11	0.77 - 1.60	1.30	0.86 - 1.95	1.43	0.99 - 2.06	1.41	0.98 - 2.04	
	Three	1.0	(11/1,106)	1.37	0.75 - 2.50	1.55	0.76 - 3.12	**1.86**	**1.02 - 3.40**	**1.90**	**1.04 - 3.47**	
	Missing, % (n)			3.3	(3,373)	7.4	(7,552)	2.2	(2,279)	2.2	(2,279)	
Caesarean section	No	18.2	(13,653/75,078)	1		1		1		1		Age, Marital status, BMI, Smok
	One	20.3	(3,494/17,240)	**1.14**	**1.10 - 1.19**	1.04	0.99 - 1.09	1.03	0.99 - 1.08	1.03	0.98 - 1.07	income), ART, Atopic Dermatit
	Two	23.7	(898/3,786)	**1.40**	**1.30 - 1.51**	**1.13**	**1.04 - 1.24**	**1.17**	**1.08 - 1.26**	**1.15**	**1.06 - 1.25**	*disease*, SLE, RA, IDDM, NIDI
	Three	27.1	(293/1,083)	**1.67**	**1.46 - 1.91**	**1.31**	**1.13 - 1.51**	**1.27**	**1.11 - 1.46**	**1.26**	**1.10 - 1.44**	Hypothyroidism, High blood pr
	Missing, % (n)			4.5	(4,611)	17.6	(17,877)	3.5	(3,583)	3.5	(3,583)	disease, *Depression*, Anxiety di:
												disease, *Gastritis*, Gastric ulcer,
												Chronic nephritis, Endometrios
												Adenomyosis, Congenital uteri

Model I Unadjusted model
Model II Adjusted model using propensity scores based on complete case analysis
Model III Adjusted model using propensity scores based on multiple imputation model
Model IV Covariates about gynecological history (underlined covariates) were added to the model III

TABLE S4 Perinatal outcome of children according to the number of previous pregnancy losses

	Number of pregnancy losses	Prevalence %, (n)		OR, 95%CI Model I		OR, 95%CI Model II		Pooled OR, 95%CI Multiple imputation model III		Pooled OR, 95%CI Multiple imputation model IV		Covariates included in pr
SFD	No	10.1	(7,634/75,225)	1		1		1		1		Age, Marital status, BMI, Smoking, Pr Collagen disease, IDDM, Anemia, Hig Congenital heart disease, Adenomyosi
	One	9.2	(1,581/17,269)	**0.89**	**0.84 - 0.94**	0.98	0.92 - 1.04	0.97	0.92 - 1.03	0.97	0.92 - 1.03	
	Two	8.4	(317/3,790)	**0.81**	**0.72 - 0.91**	0.92	0.82 - 1.04	0.94	0.83 - 1.06	0.93	0.83 - 1.05	
	Three	8.6	(94/1,089)	0.84	0.68 - 1.04	1.01	0.81 - 1.27	1.01	0.82 - 1.25	1.02	0.83 - 1.27	
	Missing, % (n)			4.3	(4,425)	10.4	(10,578)	3.3	(3,390)	3.3	(3,390)	
IUFD	No	0.4	(306/75,983)	1		1		1		1		Age, BMI, Depression, Adenomyosis
	One	0.5	(84/17,491)	1.19	0.94 - 1.52	1.10	0.82 - 1.47	1.10	0.87 - 1.41	1.10	0.86 - 1.41	
	Two	0.5	(19/3,845)	1.23	0.77 - 1.95	1.37	0.83 - 2.25	1.08	0.67 - 1.73	1.05	0.65 - 1.67	
	Three	0.8	(9/1,106)	**2.03**	**1.04 - 3.95**	**2.11**	**1.03 - 4.31**	1.68	0.86 - 3.28	1.62	0.83 - 3.38	
	Missing, % (n)			3.3	(3,373)	8.7	(8,854)	2.2	(2,279)	2.2	(2,279)	
Low birth weight<2500g	No	8.4	(6,292/75,256)	1		1		1		1		Age, Marital status, BMI, Smoking, Ed Income), ART, Previous live birth, Co NIDDM, Anemia, High blood pressur Depression, Anxiety disorders, Gastric Nephrotic syndrome, Endometriosis, I Adenomyosis, Congenital uterine ano
	One	7.9	(1,372/17,278)	0.95	0.89 - 1.01	**0.93**	**0.87 - 1.00**	0.95	0.89 - 1.01	0.94	0.89 - 1.00	
	Two	8.9	(339/3,791)	1.08	0.96 - 1.21	1.03	0.91 - 1.17	1.08	0.96 - 1.21	1.07	0.96 - 1.21	
	Three	10.1	(110/1,089)	**1.23**	**1.01 - 1.50**	**1.26**	**1.01 - 1.56**	**1.23**	**1.01 - 1.51**	1.22	1.00 - 1.49	
	Missing, % (n)			4.3	(4,385)	17.5	(17,771)	3.3	(3,351)	3.3	(3,351)	
Male	No	51.3	(38,796/75,668)	1		1		1		1		Dysautonomia
	One	51.7	(8,994/17,386)	1.02	0.99 - 1.05	1.02	0.98 - 1.05	1.02	0.99 - 1.05	1.02	0.99 - 1.05	
	Two	51.7	(1,975/3,823)	1.02	0.95 - 1.08	1.02	0.95 - 1.09	1.02	0.95 - 1.09	1.02	0.95 - 1.09	
	Three	48.6	(534/1,098)	0.90	0.80 - 1.01	0.90	0.80 - 1.02	0.90	0.80 - 1.02	0.90	0.80 - 1.02	
	Missing, % (n)			3.8	(3,823)	5.1	(5,141)	2.7	(2,771)	2.7	(2,771)	
Apgar score <7 at 5 minute	No	0.6	(443/71,370)	1		1		1		1		Age, BMI, Smoking, Household incor birth, IDDM, NIDDM, High blood pre Uterine fibroids, Adenomyosis
	One	0.8	(128/16,441)	**1.26**	**1.03 - 1.53**	**1.29**	**1.03 - 1.61**	**1.23**	**1.01 - 1.50**	1.21	0.99 - 1.48	
	Two	0.6	(22/3,615)	0.98	0.64 - 1.51	0.87	0.52 - 1.44	0.93	0.60 - 1.44	0.92	0.60 - 1.43	
	Three	0.6	(6/1,040)	0.93	0.41 - 2.08	0.37	0.09 - 1.49	0.89	0.39 - 2.02	0.85	0.38 - 1.91	
	Missing, % (n)			9.2	(9,332)	21.2	(21,551)	8.2	(8,378)	8.2	(8,378)	
pH<7.1	No	1.2	(730/63,024)	1		1		1		1		ART, Previous live birth, NIDDM, An Adenomyosis
	One	1.2	(178/14,546)	1.06	0.90 - 1.25	1.17	0.99 - 1.38	1.17	0.99 - 1.39	1.17	0.99 - 1.38	
	Two	1.1	(36/3,190)	0.97	0.70 - 1.36	1.13	0.80 - 1.59	1.13	0.80 - 1.59	1.13	0.80 - 1.58	
	Three	1.2	(11/932)	1.02	0.56 - 1.86	1.14	0.61 - 2.13	1.21	0.66 - 2.21	1.21	0.66 - 2.21	
	Missing, % (n)			19.8	(20,106)	20.8	(21,147)	19.0	(19,342)	19.0	(19,342)	
Congenital anomaly	No	12.2	(9,077/74,241)	1	(20,107)	1		1		1		Age, BMI, Education, (Household inc disease, IDDM, Gestational diabetes, H Hypothyroidism, High blood pressure Congenital heart disease, Gastritis, Irri Uterine fibroids, Congenital uterine ar
	One	12.6	(2,143/17,408)	1.03	0.98 - 1.09	1.00	0.95 - 1.06	1.01	0.96 - 1.06	1.01	0.96 - 1.06	
	Two	13.1	(492/3,751)	1.08	0.98 - 1.20	1.01	0.91 - 1.12	1.04	0.94 - 1.14	1.03	0.93 - 1.14	
	Three	12.4	(134/1,079)	1.02	0.85 - 1.22	0.95	0.78 - 1.15	0.95	0.79 - 1.14	0.94	0.78 - 1.13	
	Missing, % (n)			5.6	(5,679)	17.6	(17,937)	4.6	(4,673)	4.6	(4,673)	
Aneuploidy	No	0.2	(145/75,383)	1		1		1		1		Age, Previous live birth, Uterine fibro
	One	0.2	(39/17,299)	1.17	0.82 - 1.67	0.97	0.68 - 1.39	0.97	0.68 - 1.38	0.95	0.67 - 1.36	
	Two	0.2	(8/3,796)	1.10	0.54 - 2.24	0.81	0.39 - 1.65	0.78	0.38 - 1.59	0.78	0.38 - 1.60	
	Three	0.2	(2/1,089)	0.96	0.24 - 3.86	0.62	0.15 - 2.54	0.60	0.15 - 2.43	0.59	0.14 - 2.39	
	Missing, % (n)			4.2	(4,231)	7.8	(7,972)	3.1	(3,193)	3.1	(3,193)	
